# Difluoromethylornithine (DFMO) and AMXT 1501 inhibit capsule biosynthesis in pneumococci

**DOI:** 10.1038/s41598-022-16007-7

**Published:** 2022-07-12

**Authors:** Moses B. Ayoola, Leslie A. Shack, Jung Hwa Lee, Juhyeon Lim, Hyungjin Eoh, Edwin Swiatlo, Otto Phanstiel, Bindu Nanduri

**Affiliations:** 1 Department of Comparative Biomedical Sciences, College of Veterinary Medicine, Mississippi State University, Mississippi State, MS 39762 USA; 2 Zilkha Neurogenetic Institute, University of Southern California, Los Angeles, CA USA; 3 Section of Infectious Diseases, Southeast Louisiana Veterans Health Care System, New Orleans, LA 70112 USA; 4 Department of Medical Education, College of Medicine, University of Central Florida, Orlando, FL USA; 5 Institute for Genomics, Biocomputing and Biotechnology, Mississippi State University, Mississippi State, MS 39762 USA

**Keywords:** Bacteria, Infectious-disease diagnostics

## Abstract

Polyamines are small cationic molecules that have been linked to various cellular processes including replication, translation, stress response and recently, capsule regulation in *Streptococcus pneumoniae* (Spn, pneumococcus). Pneumococcal-associated diseases such as pneumonia, meningitis, and sepsis are some of the leading causes of death worldwide and capsule remains the principal virulence factor of this versatile pathogen. α-Difluoromethyl-ornithine (DFMO) is an irreversible inhibitor of the polyamine biosynthesis pathway catalyzed by ornithine decarboxylase and has a long history in modulating cell growth, polyamine levels, and disease outcomes in eukaryotic systems. Recent evidence shows that DFMO can also target arginine decarboxylation. Interestingly, DFMO-treated cells often escape polyamine depletion via increased polyamine uptake from extracellular sources. Here, we examined the potential capsule-crippling ability of DFMO and the possible synergistic effects of the polyamine transport inhibitor, AMXT 1501, on pneumococci. We characterized the changes in pneumococcal metabolites in response to DFMO and AMXT 1501, and also measured the impact of DFMO on amino acid decarboxylase activities. Our findings show that DFMO inhibited pneumococcal polyamine and capsule biosynthesis as well as decarboxylase activities, albeit, at a high concentration. AMXT 1501 at physiologically relevant concentration could inhibit both polyamine and capsule biosynthesis, however, in a serotype-dependent manner. In summary, this study demonstrates the utility of targeting polyamine biosynthesis and transport for pneumococcal capsule inhibition. Since targeting capsule biosynthesis is a promising way for the eradication of the diverse and pathogenic pneumococcal strains, future work will identify small molecules similar to DFMO/AMXT 1501, which act in a serotype-independent manner.

## Introduction

Polyamines such as putrescine, spermidine, spermine and cadaverine, are positively charged biogenic amines that regulate a number of cellular processes via their interactions with negatively charged molecules such as nucleic acids, proteins, and phospholipids^1^. Polyamines are ubiquitous in nature and their metabolism is well characterized in both eukaryotic and prokaryotic systems, specifically in bacteria including pathogenic bacteria. Polyamine biosynthesis is described in human, and bacterial pathogens such as *Escherichia coli*, *Pseudomonas aeruginosa*, *Campylobacter jejuni*, *Salmonella enterica*, and host of other biological systems^2–4^. Pneumococcal genome annotation includes polyamine synthesis and transport genes. However, organisms such as *Enterococcus faecalis* and *Staphylococcus aureus* have either incomplete or lack polyamine biosynthesis pathways, respectively^5^. Polyamines are critical for eukaryotic cell proliferation and the canonical pathway for polyamine biosynthesis in both eukaryotes and prokaryotes involves sequential actions of arginase and ornithine decarboxylase (ODC) that convert arginine to ornithine, and ornithine to putrescine, respectively. Alternatively, putrescine could be synthesized from arginine via agmatine and *N*-carbamoylputrescine through sequential enzymatic reactions catalyzed by arginine decarboxylase, agmatine deiminase and carbon–nitrogen hydrolase family proteins respectively. Addition of the aminopropyl moiety of decarboxylated *S*-adenosylmethionine to putrescine and spermidine by spermidine/spermine synthase generates spermidine and spermine, respectively. ODC is known to catalyze the first and rate limiting step in polyamine synthesis in eukaryotes and decarboxylases like ODC are pyridoxal-5′-phosphate-dependent (PLP) enzymes. When treated with α-difluoromethylornithine (DFMO), an irreversible inhibitor of ODC, cells are typically depleted of putrescine and spermidine^6^.

DFMO has a 40-year history in clinical research, mostly in anti-cancer studies^7,8^. The combined therapy of DFMO and AMXT 1501, a novel polyamine transport inhibitor, was recently shown to be more effective in the treatment of neuroblastoma^9^ and in delaying the progression of autoimmune encephalomyelitis^10^ than single therapies.

Beyond cancer, DFMO’s ability to inhibit cell proliferation has shown diverse applications in the treatment of other diseases including African trypanosomiasis^11^. DFMO also has FDA approval in the form of Vaniqa cream to inhibit unwanted facial hair growth in women^12,13^. In addition, DFMO was shown to inhibit the growth of fungi such as *Colletotrichum truncatum* and *Cochliobolus carbonum*, which are economically important pathogens of soybean and sweet corn, respectively^14^. Despite the numerous reports on the role of DFMO in eukaryotes, there are relatively few reports on its application in prokaryotes, especially in bacterial physiology and pathogenesis.

Experimental evidence shows that DFMO treatment induces oxidative stress in *H. pylori* and reduces the toxin-associated virulence factor in this pathogen that affects the human digestive tract^15^. DFMO treatment of *Streptococcus pneumoniae* (pneumococcus, Spn) serotype 3 (WU2) that harbors a deletion in *potD*, a gene that encodes polyamine transport binding domain protein, resulted in impaired growth^16^. Although, deletion of *potD* alone had no impact on growth in WU2 strain. However, deletion of arginine decarboxylase (ADC/*speA*) that synthesizes agmatine, an intermediate from the putrescine/spermidine biosynthesis pathway in serotype 4 pneumococci results in the inhibition of capsular polysaccharide (CPS) synthesis^17,18^. Exogenous supplementation of agmatine restores capsule, indicating that reduced intracellular levels of this polyamine due to impaired synthesis are critical for CPS regulation^18^. The capsule is the principal virulence factor in pneumococcus, the most common bacterial etiology of otitis media, sepsis, and community-acquired pneumonia globally^19^. Capsule is necessary for escape from host opsonophagocytosis^20^ and the basis for the classification of the known 100 serotypes based on distinct polysaccharide compositions^21^.

Apart from inhibition of ODC activity that impedes putrescine synthesis, DFMO has also been shown to inhibit arginine decarboxylase (ADC) activity^22^ that synthesizes agmatine. Therefore, in this study, we hypothesized that inhibition of polyamine biosynthesis by DFMO could impact capsule synthesis. Using multiple pneumococcal serotypes that follow the conventional Wzy-dependent (2, 4 and 19F) and the uncommon synthase- dependent pathway (serotype 3) for capsule biosynthesis^23^, we show that DFMO inhibits CPS synthesis in Spn. Metabolomic analysis of DFMO-treated Spn identified reduced precursors and carbohydrates that constitute the CPS repeat unit, similar to our earlier reports on the inhibition of CPS by genetic manipulation, i.e., via deletion of the *speA* gene^24^. We also show that agmatine supplementation restores capsule in DFMO-treated pneumococci. Thus, polyamine-mediated regulation of CPS is observed in both the chemical approach (this study with DFMO) and the genetic (∆*speA*) approach to impair polyamine synthesis. Inhibition of polyamine transport with AMXT 1501 had no impact on CPS on majority of pneumococcal serotypes and we did not identify synergistic effects of DFMO and AMXT 1501 on CPS synthesis. This result suggests that polyamine transport did not play a key role in capsule formation. However, serotype independent chemical inhibition of polyamine synthesis by DFMO (with its adverse effect on CPS identified here) holds promise for developing novel therapeutics that could incapacitate most or all the virulent pneumococcal serotypes, by modulating polyamine homeostasis.

## Materials and methods

### Characterization of α-difluoromethylornithine (DFMO) impact on pneumococcal growth

*S. pneumoniae* serotypes 4 (TIGR4), 2 (D39), 19F (EF3030) and 3 (WU2) were cultured in Todd Hewitt broth supplemented with yeast extract (THY). Supplementation with DFMO ranging from 1.1 to 549 mM was carried out in triplicate in a 96-well plate and incubated at 37 °C and 5% CO_2_ in a Cytation™ 5 cell imaging multi-mode reader (BioTek, Winooski, VT). The change in optical density at 600 nm (OD_600_) was measured every hour for 24 h and used to determine the minimum inhibitory concentration (MIC) of DFMO. The optical density data was analyzed using GrowthRates 4.0 software^25^. To determine whether DFMO at sub-MIC is bacteriostatic or lethal to pneumococcal cells, all strains were cultured in the presence or absence of 1/2 MIC DFMO. OD_600_ was measured every hour and CFU were enumerated on a blood agar plate (BAP) every 2 h over an 8 h incubation period.

### Impact of DFMO and AMXT 1501 on pneumococcal capsule

*S. pneumoniae* strains were cultured with DFMO and AMXT 1501 at varying final concentrations of 1/2, 1/4, and 1/8 MIC to OD_600_ 0.2–0.3 either individually or together to estimate any additive or synergistic effects. Capsular polysaccharides (CPS) were extracted and quantified in triplicate, as described previously^26^ with slight modification. Briefly, CPS was extracted in a lysis buffer (4% deoxycholate, 50 µg/mL DNAse I and 50 µg/mL RNAse A) at 37 °C for 10 min and centrifuged at 18,000×*g* for 10 min. The supernatant (2 µL) was spotted on a 0.2-µm-pore-size nitrocellulose membrane (Thermo Fisher Scientific, Waltham, MA, USA) and oven dried at 60 °C for 15 min. The membranes were blocked and incubated with different types of antisera for capsule detection: antisera type- 4 (for TIGR4), 2 (for D39), polyclonal (for EF3030) and 3 (for WU2) (Cedarlane, Burlington, NC, USA). A conjugated polyclonal goat anti-rabbit IgG-HRP (Agilent Technologies, Santa Clara, CA, USA) was used for enhanced chemiluminescence (ECL) detection in all the strains (Thermo Fisher Scientific, Waltham, MA, USA) and scanned using a ChemiDoc XRS+ with Image Lab software (Bio-Rad, Hercules, CA, USA). Densitometry analysis of the immunoblot was done with NIH ImageJ software^27^.

### Agmatine supplementation of DFMO-treated pneumococci

We determined the impact of varying concentrations of DFMO (1/8 to 1/2 MIC) on CPS in all serotypes (2, 4, 19F, and 3) used in this study. However, agmatine (1/4 MIC, 20 mM) supplementation with potential to restore loss of capsule was performed with serotype 2 (D39) which is more sensitive to DFMO (1/8 MIC, 34 mM). D39 was treated with 1/8 MIC DFMO alone, 1/4 MIC agmatine (Agm) alone, a combination of 1/8 MIC DFMO and 1/4 MIC Agm, and a control without DFMO or Agm. CPS was extracted as described in the previous section and immunoblot analysis with type 2 antisera was performed to detect CPS semi-quantitatively.

### Metabolomics of DFMO-treated pneumococci

*S. pneumoniae* TIGR4 (10 mL, n = 5) was grown in the absence or presence of DFMO (137 mM, 1/2 MIC) to an OD_600_ of 0.4–0.5. This was done to directly compare possible changes in intracellular concentrations of polyamines and capsule precursors as a result of DFMO inhibition of polyamine synthesis to the earlier report where polyamine synthesis was affected via gene deletion in TIGR4^24^. D39 on the other hand was cultured in the absence or presence of impactful concentrations of DFMO (1/8 MIC, 34 mM), AMXT 1501 (1/2 MIC, 7.4 µM), agmatine (1/4 MIC, 20 mM), and combination of either DFMO or AMXT 1501 and agmatine to OD_600_ 0.4–0.5. Cells were harvested (5000×*g*, 10 min, 4 °C), re-suspended in extraction solvent [40% acetonitrile, 40% methanol, 20% water with formic acid (0.1 M)] and transferred to bead-beater tubes (MP Biomedicals, Irvine, CA, USA). Cells were lysed using a FastPrep-24™ Classic (45 s, 6.5 m/s, RT, 3 times) (MP Biomedicals, Irvine, CA, USA) and incubated on ice (5 min after each spin). TIGR4 lysates were clarified (6000×*g*, 5 min, 4 °C), and metabolite differentiation and detection were performed using published protocols^28,29^. Liquid chromatography-mass spectrometry-based (LC–MS) metabolomic analysis was performed with an Agilent Accurate Mass 6230 TOF coupled with an Agilent 1290 LC system using a Cogent Diamond Hydride Type C column. Briefly, the mobile phase consisted of the following: solvent A (ddH2O with 0.2% formic acid) and solvent B (acetonitrile with 0.2% formic acid). The gradient used was as follows: 0–2 min, 85% B; 3–5 min, 80% B; 6–7 min, 75% B; 8–9 min, 70% B; 10–11.1 min, 50% B; 11.1–14 min 20% B; 14.1–24 min 5% B followed by a 10 min re-equilibration period at 85% B at a flow rate of 0.4 mL/min. Mass axis dynamics was calibrated by continuous infusion of a reference mass solution using an isocratic pump. This configuration achieved mass errors of 5 ppm, mass resolution ranging from 10,000 to 25,000 (over m/z 121–955 atomic mass units), and 5 × log10 dynamic range. Metabolite identities were searched for using a mass tolerance of < 0.005 Da. Metabolite concentrations from the 5 biological replicates were normalized to biomass based on measurement of residual peptide content in individual samples using the Pierce BCA Protein Assay kit (Thermo Fisher Scientific, Waltham, MA, USA). Data were analyzed using Profinder B.07.00 software (Agilent Technologies, Santa Clara, CA, USA). Extracted molecular features detected in the mass analyzer were identified using accurate mass values and used to generate empirical molecular formulae using an in-house metabolite database that included known intermediates, amino acid precursors and polyamines and their derivatives. Statistical analysis of metabolite peak intensity data was performed using MetaboAnalyst 4.0^30^. Data were normalized (quantile), log transformed and significant fold change between WT and DFMO-treated WT were identified by Student’s *t*-test (p ≤ 0.05).

To measure the polyamines, glucose and glucuronic acid in D39, TSQ Quantum Access triple-quadrupole tandem mass spectrometer (Thermo Fisher Scientific, Waltham, MA, USA) equipped with acquity UPLC system (Waters, Milford, MA, USA) was operated in positive and negative ion mode. Chromatographic separation was performed on Cogent Diamond Hydride HPLC column (2.1 mm × 150 mm, 4 μm) coupled with Congent Diamond Hydride HPLC Guard column (2.0 mm × 10 mm, 4 μm). The flow rate was 500 µL/min, the sample injection volume was 10 µL, and the column temperature was at 40 °C. The mobile phase A was composed of water with 0.1% v/v formic acid, and mobile phase B was composed of acetonitrile with 0.1% v/v formic acid. The gradient condition was 0 min (5% A, 95% B), 1 min (5% A, 95% B), 4.0 min (30% A, 70% B), 6 min (95% A, 5% B), 6.5 min (95% A, 5% B), 7 min (5% A, 95% B), and 10 min (5% A, 95%B). The total run time was 10 min, and the column eluate directed into the mass spectrometer using an electrospray ionization interface in positive and negative modes. The MS conditions were set as follow: spray voltage = 3500 V, vaporizer temperature = 350 °C, sheath gas = 25 psi, auxiliary gas = 10 psi, and capillary temperature = 350 °C. Samples were run in selected reaction monitoring (SRM) mode, and the optimized MS/MS parameters for ionization are given in Table 1. Both Spermidine-d_8_ and Glucose-d_7_ were used as an internal standard (IS) in positive and negative modes. Optimum collision energy and S-lenses conditions were determined for each compound by using auto-tune software for each analyte by post-column infusion of the individual compounds into a 50% A/50% B blend of the mobile phase pumped at a flow rate of 0.5 mL/min. Xcalibur software was employed for data acquisition and processing.

**Table 1:** Parameter for different selected reaction monitoring acquisition.

Analyte	Ion mode	SRM transition (*m/z*)	Collision energy
**Polyamines**			
Arginine	[M + H]^+^	175.1 > 70.5	22
Lysine	[M + H]^+^	147.1 > 84.4	14
Ornithine	[M + H]^+^	133.2 > 116.3	5
Agmatine	[M + H]^+^	131.2 > 72.4	14
Cadaverine	[M + H]^+^	103.2 > 86.4	5
Putrescine	[M + H]^+^	89.2 > 72.5	5
Spermidine	[M + H]^+^	146.1 > 112.3	12
Spermine	[M + H]^+^	203.2 > 112.3	19
*N*-carbamoyl putrescine	[M + H]^+^	132.1 > 115.2	7
*N-*acetyl spermidine	[M + H]^+^	245.2 > 100.2	19
Spermidine-*d*_8_ (IS)	[M + H]^+^	154.2 > 80.5	16
**Sugars**			
Glucose	[M + HCOO]^−^	225.1 > 178.8	10
Glucuronic acid	[M − H]^−^	193.1 > 112.8	14
Glucose-*d*_7_ (IS)	[M + HCOO]^−^	232.1 > 186.2	8

### Inhibition of decarboxylase activity by DFMO and DFMA

SP_0916 in TIGR4 encodes an ADC that has some minimal ODC and lysine decarboxylase (LDC) activities^18^. We determined the impact of DFMO (ODC/ADC inhibitor) and α-difluoromethylarginine (DFMA, an ADC inhibitor) on the decarboxylase activities of SP_0916. BL21(DE3) cells expressing recombinant His tag SP_0916 were lysed with B-PER reagent buffer (Thermo Fisher Scientific, Waltham, MA, USA), lysate separated by loading unto HisPur Cobalt Spin Column (Thermo Fisher Scientific, Waltham, MA, USA), desalted using Sephadex G-25 PD-10 column (GE Healthcare, Chicago, IL, USA), and protein concentration estimated using the Pierce BCA Protein Assay kit (Thermo Fisher Scientific, Waltham, MA, USA).

SP_0916-encoded protein (0.926 µmol L^−1^) was mixed with 10 mM substrate (arginine, lysine or ornithine) in the reaction mixture (500 µL reaction volume, 50 mM Tris–HCl, pH 8.0) that contains 2.5 mM MgSO_4_ and 0.6 mM PLP. DFMO was diluted in water to 3–900 mM and added to the reaction mixture after 5 min preincubation at 37 °C in the dark. DFMA stock was made in a similar manner but was diluted in water to 0.3–1000 µM. The enzyme and inhibitor were incubated at 37 °C for 15 min in the dark and the reaction terminated with 0.3 M perchloric acid. After incubation on ice for 15 min, the samples were neutralized with 25 µL of 10 N KOH and extracted with 1 mL of 1-butanol. The organic layer was dried under nitrogen gas and reconstituted with 100 µL of water containing Spermidine-d_8_ and Glucose-d_7_ as an internal standard. Analysis of the extracts was performed on a Surveyor LC–MS system. 5 µL of reconstitution was injected to the LC–MS system for detection of the products (agmatine, cadaverine or putrescine). We used internal standards to normalize and determine the peak intensity (area ratio) for each metabolite detected. Enzyme activity without inhibitor is referred to as a control activity. All experiments were performed in triplicate. The concentration of inhibitor that inhibited 50% of the control activity (IC_50_ value) was determined by varying the concentration of the indicated DFMO or DFMA and measuring the decarboxylase activity. Sigma Plot v. 12 was used to fit the curve through the points and IC_50_ values were interpolated from the fitted curve.

## Results

### Impact of DFMO on pneumococcal growth

**Figure 1:**
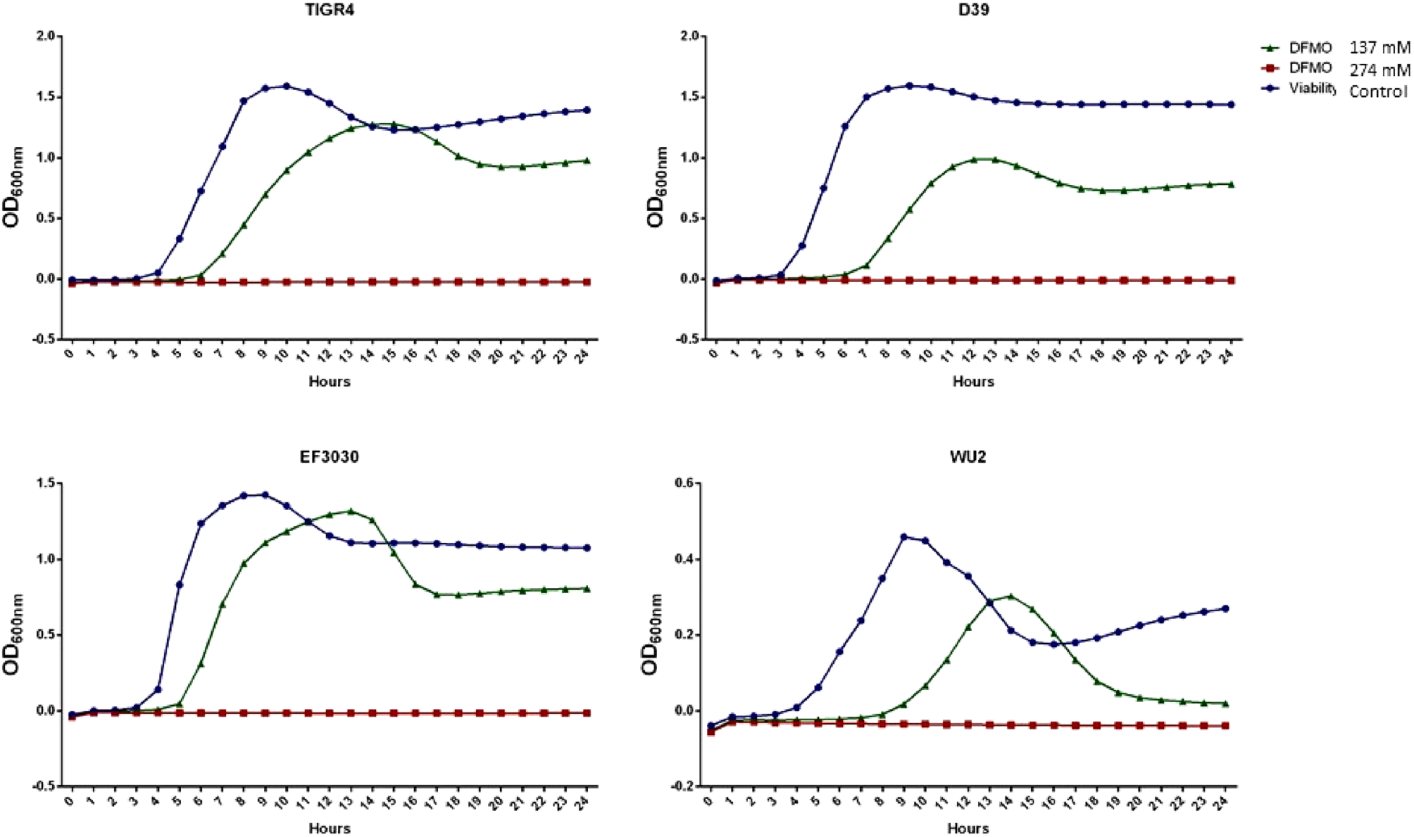
Susceptibility of pneumococci to DFMO. Growths of TIGR4, D39, EF3030 and WU2 strains were measured with DFMO (137 and 274 mM) and without (viability control) in a plate reader at 1 h intervals for 24 h.

DFMO is an irreversible inhibitor of ODC and polyamine synthesis in eukaryotic systems. DFMO can be either bacteriostatic or bactericidal. Here, the susceptibility of four different pneumococcal serotypes to DFMO was examined to determine the growth permissible concentration. Our results show that the minimum inhibitory concentration (MIC) for all four serotypes is the same, at 274 mM (Fig. 1). At 1/2 MIC DFMO (137 mM), D39 and WU2 exhibited a longer lag phase and a lower maximal O.D compared to controls (Fig. 1). However, analysis of growth curves with GrowthRates software did not identify any significant differences between the growth rate of all serotypes tested in the presence or absence of DFMO (data not shown).

**Figure 2:**
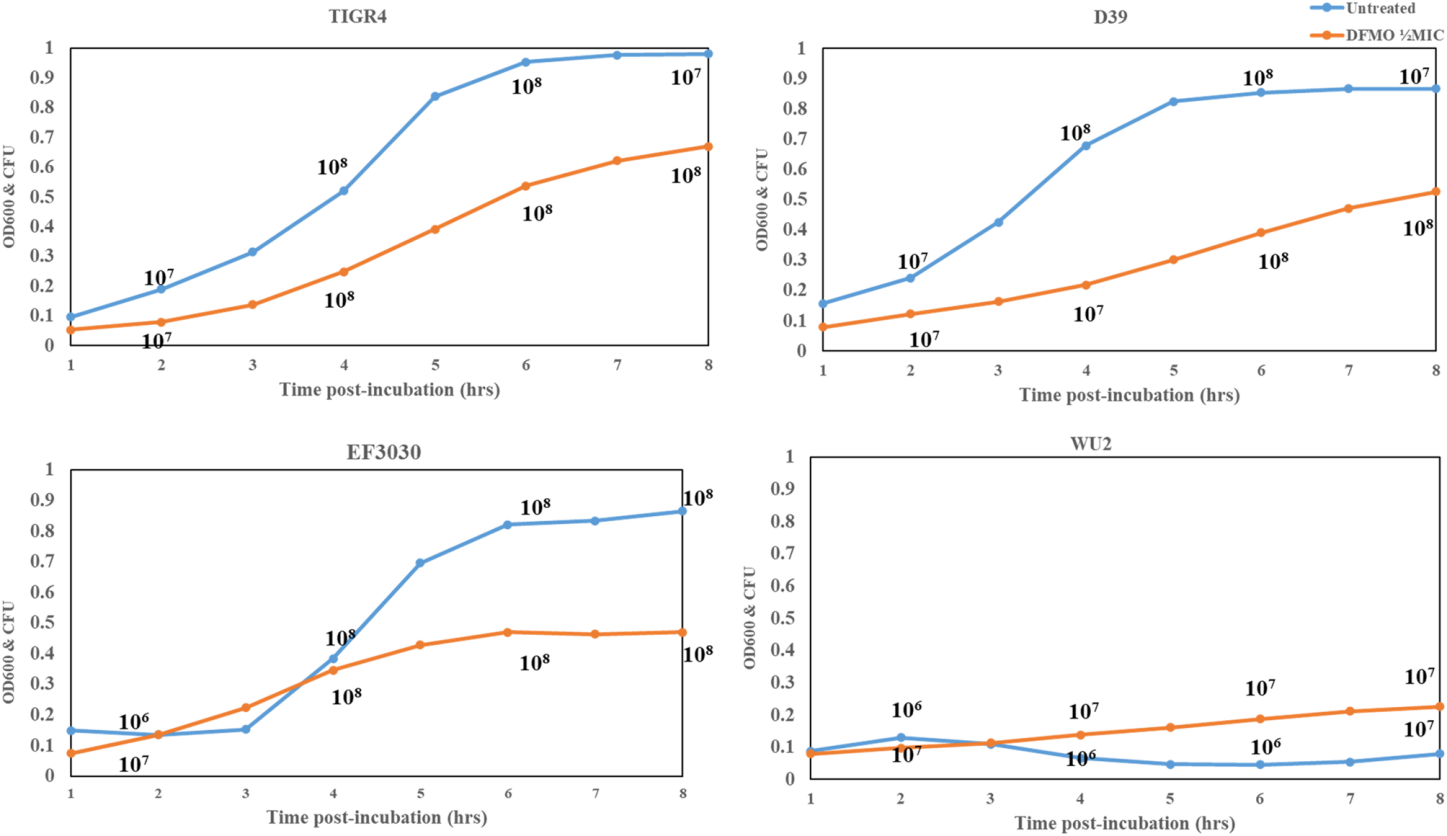
Impact of DFMO on pneumococci viability/replication. Optical density vs. colony forming unit (CFU/mL) of TIGR4, D39, EF3030 and WU2 treated with DFMO (orange, 1/2 MIC, 137 mM) with respect to the untreated (blue) show some bacteriostatic effect of DFMO in all serotypes except WU2.

Since the focus of this work is to identify the impact of DFMO on CPS of viable bacterial cells, we determined the impact of 1/2 MIC DFMO on viability. Viability curves at 1/2 MIC DFMO indicate some bacteriostatic effect of DFMO on all the pneumococci strains with the exception of WU2 (Fig. 2).

### DFMO and AMXT 1501 inhibit expression of CPS

**Figure 3:**
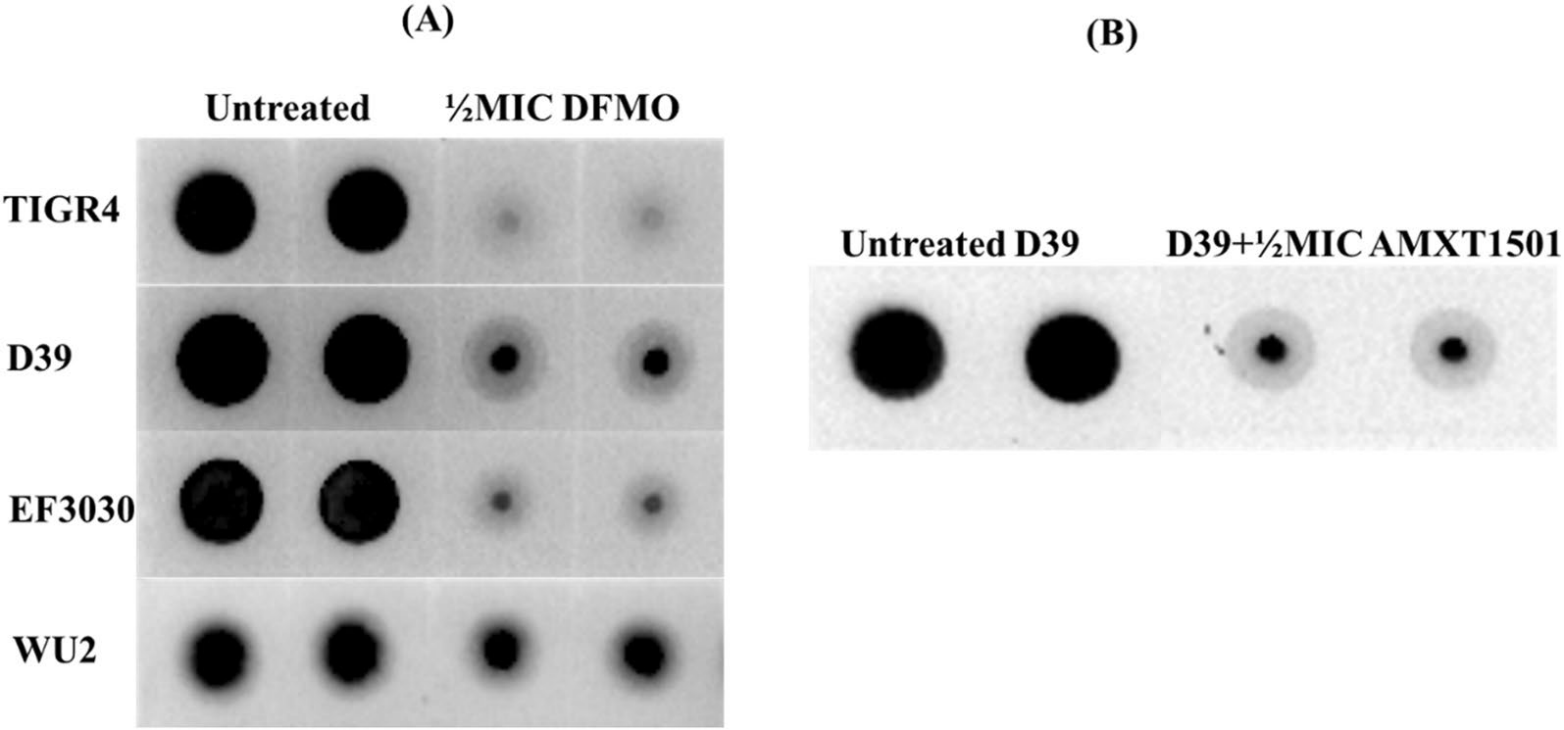
DFMO inhibition of the capsule synthesis is serotype independent (**A**) while loss of capsule by AMXT 1501 treatment is observed only in pneumococcal serotype 2, D39 (**B**). Immunoblots for total CPS were performed with serotype-specific antibody (type: 4 for TIGR4, 2 for D39, polyclonal serum for EF3030, and 3 for WU2) to estimate total CPS in DFMO treated and untreated pneumococcal serotypes.

Here, we examined the impact of DFMO and AMXT 1501 (a polyamine transport inhibitor), on capsule biosynthesis. Our results show that total CPS in TIGR4, D39, EF3030, and WU2 is significantly reduced (Fig. 3A) at a non-lethal concentration of DFMO (1/2 MIC, 137 mM). AMXT 1501 had an impact on CPS only in D39 (Fig. 3B). Densitometry analysis of immunoblots indicates approximately 85, 71, 84 and 39% less capsule in TIGR4, D39, EF3030 and WU2, respectively, compared to untreated controls, while there was 61% less capsule in D39 treated with 1/2 MIC AMXT 1501 (7.4 µM). These results show that DFMO inhibits capsule biosynthesis in a serotype-independent manner, although the inhibition is to a relatively smaller proportion in serotype (WU2) that follows synthase mechanism of CPS synthesis. Concentrations of DFMO less than 137 mM failed to inhibit CPS in serotypes 4, 19F and 3. However, CPS synthesis was inhibited in D39 at 1/8 MIC (34 mM) DFMO (data now shown). These results suggest that there may be some serotype differences in susceptibility to polyamine inhibitors.

### Impact of DFMO on pneumococcal metabolome

#### TIGR4 metabolites

**Figure 4:**
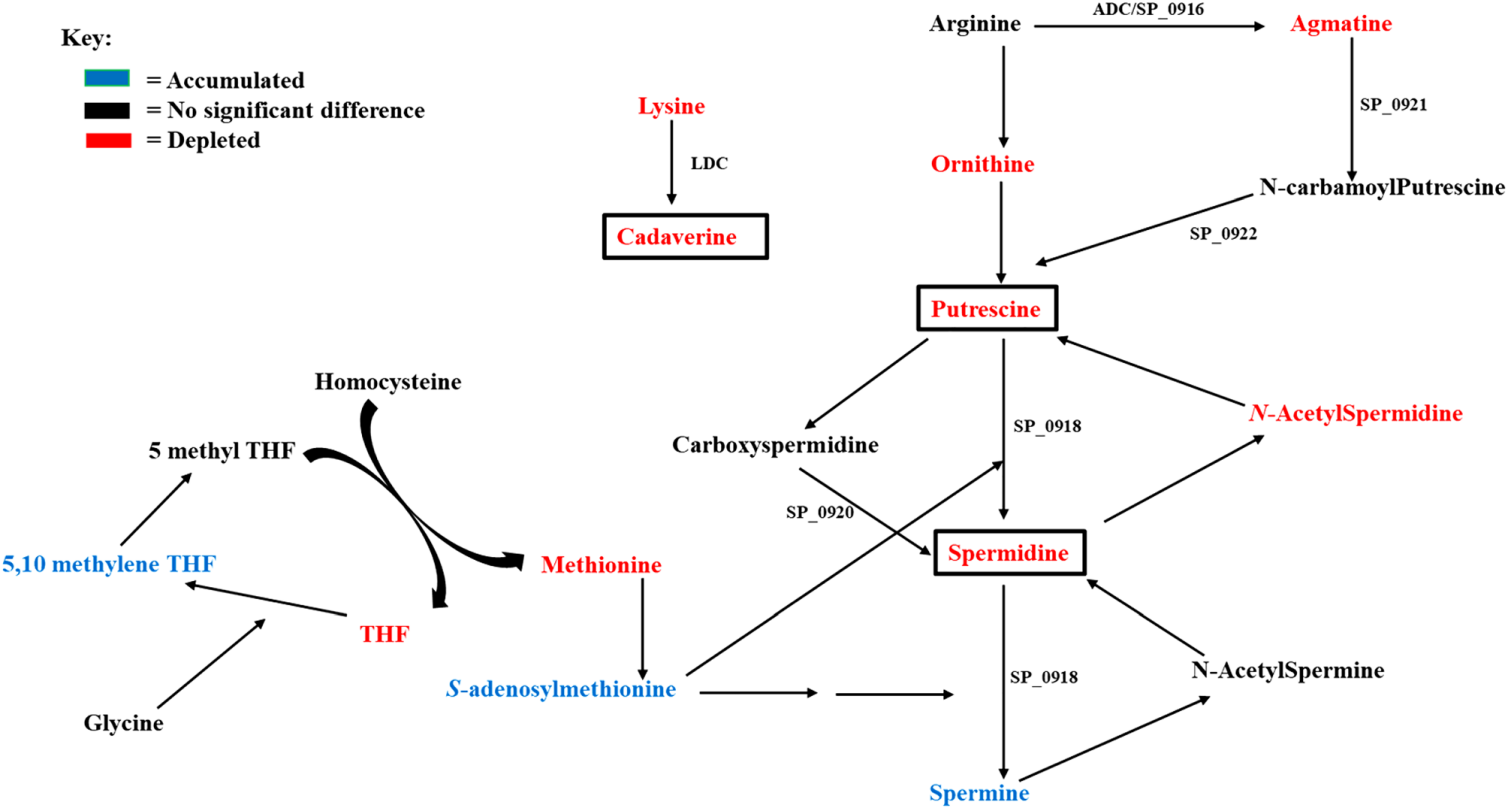
Polyamine biosynthesis pathway and metabolites in TIGR4 as impacted by DFMO. Metabolites in red are depleted, blue are accumulated, while black are not significantly impacted.

To identify metabolic pathways targeted by DFMO in TIGR4, we performed mass spectrometry-based metabolomic analysis of TIGR4 and TIGR4 treated with 1/2 MIC DFMO. We identified significant changes in the intracellular concentrations of 36 metabolites (Supplementary Table 1). DFMO treatment resulted in significant reduction in the polyamines putrescine, spermidine, and cadaverine (Fig. 4). In addition, the observed reduced level of *N*-acetylspermidine was consistent with the depletion of spermidine. Furthermore, a significant accumulation of *S*-adenosylmethionine (SAM) was observed. SAM in its decarboxylated form is an aminopropyl donor for the conversion of putrescine to spermidine. Accumulation of 5,10-methylenetetrahydrofolate indicates that homocysteine is not being converted to methionine and tetrahydrofolate (THF) which led to their depletion. The amino acid methionine is the precursor of SAM, and the observed levels of 5,10-methylene and SAM suggest a feedback inhibition of the synthesis of methionine by SAM. The observed reduced level of agmatine, the product of arginine decarboxylation, corroborates the fact that DFMO not only inhibits canonical pathway for polyamine biosynthesis via ODC, but also the alternative pathway catalyzed by ADC. Arginine, the precursor amino acid in the alternative pathway for putrescine/spermidine synthesis is not affected, while ornithine is depleted. Both lysine and cadaverine, i.e., the precursor and product of LDC, are reduced.

**Figure 5:**
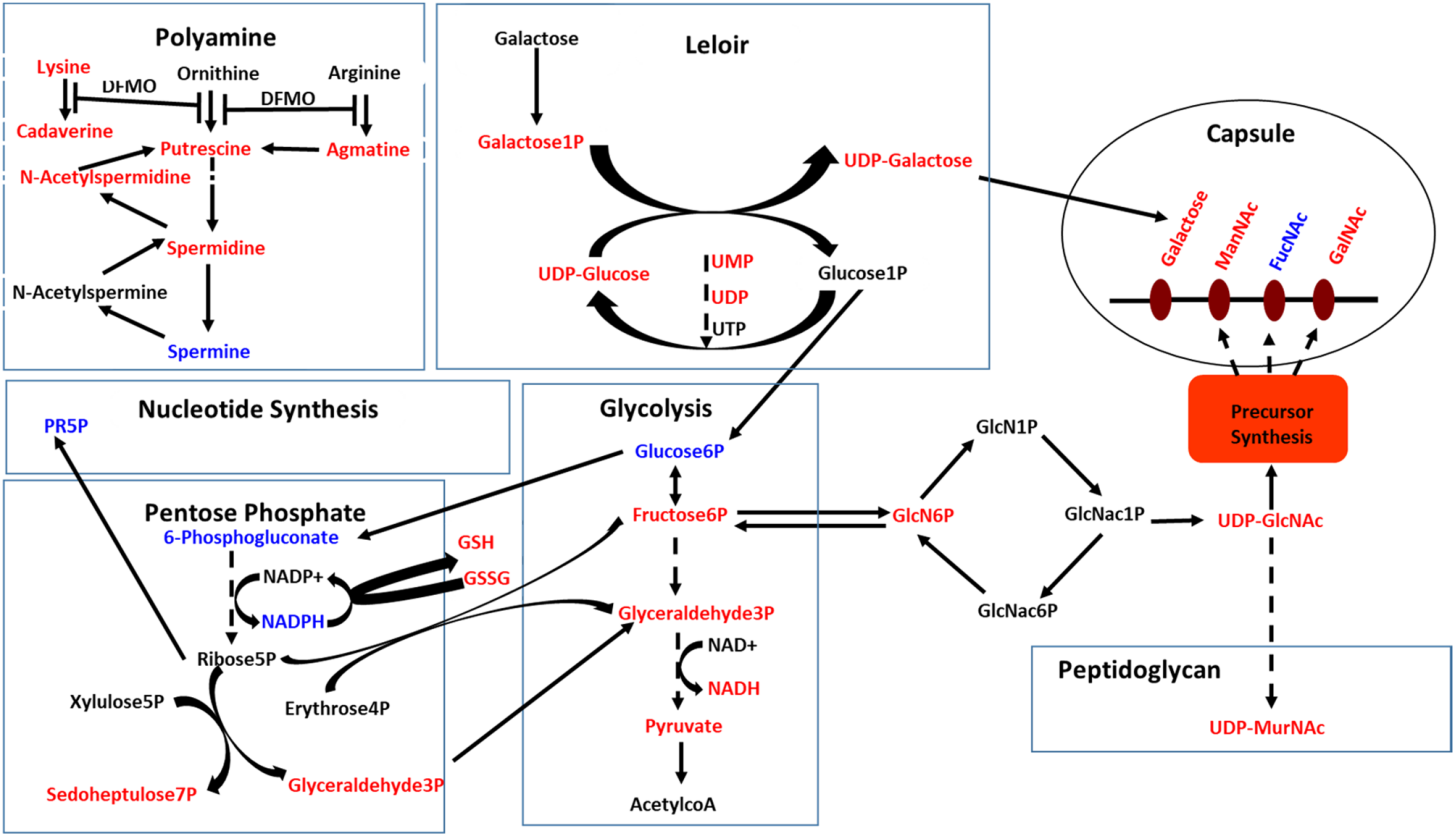
DFMO impacts polyamine synthesis, carbohydrate metabolism, capsule biosynthesis, and oxidative stress responses in pneumococci. Significant accumulation of metabolites is shown in blue, depletion is shown in red, while black represents no detected change. Multi-step reactions are represented by a broken arrow. DFMO treatment inhibits agmatine, cadaverine, putrescine and spermidine synthesis. UDP-Galactose production via Leloir pathway is inhibited. Impaired glycolytic pathway activity results in reduced glucosamine 6-phosphate (GlcN6P) and ultimately UDP-N-acetylglucosamine (UDP-GlcNAc), a precursor for sugars in serotype 4 CPS repeat unit (open oval). Altered UDP-GlcNAc also impacts UDP-*N*-acetylmuramic acid (UDP-MurNAc) availability for peptidoglycan assembly (open rectangle) which is the site of attachment of capsule. Redirection of glucose via glucose 6-phosphate from glycolytic pathway to pentose phosphate pathway, and subsequent increase in NADPH and 5-phospho-α-d-ribose 1-diphosphate (PRPP) are consistent with increased oxidative stress response and repair in DFMO treated cells.

Changes in the intracellular levels of polyamines appears to repress the Leloir pathway, as seen by reduction in the concentrations of galactose 1-phosphate, UDP-galactose, and UDP-glucose (Fig. 5). UDP-galactose is the donor of galactose, one of the four repeating unit sugars in serotype 4 CPS. Significant accumulation of Glucose 6-phosphate and depletion of fructose 6-phosphate, glyceraldehyde 3-phosphate, pyruvate and NADH indicate impaired glycolysis with possible upregulation of pentose phosphate pathway (PPP). Glucose 6-phosphate is the direct and pivotal intermediate of glucose that is at the intersection of glycolysis and PPP, while fructose 6-phosphate links glycolysis to nucleotide sugar biosynthesis (Fig. 5). Changes in the Leloir and glycolysis pathways leads to the depletion of glucosamine 6-phosphate (GlcN6P) and UDP-*N*-acetylglucosamine (UDP-GlcNAc) in nucleotide sugar biosynthesis. UDP-GlcNAc is the substrate of epimerase for the formation UDP-*N*-acetylgalactosamine, UDP-*N*-acetylmannosamine and UDP-*N*-acetylfucosamine which are the precursors for the remaining three repeat unit sugars in serotype 4 CPS. Our results show significant depletion of UDP-*N*-acetylgalactosamine and UDP-*N*-acetylmannosamine with a modest accumulation of UDP-*N*-acetylfucosamine. Significant depletion of UDP-*N*-acetylmuramic acid, precursor for peptidoglycan cell wall to which capsule is attached, and UMP/UDP that provide UDP for nucleotide sugars was also observed. Accumulated 6-phosphogluconate led to increased levels of NADPH in PPP, and 5-phospho-α-d-ribose 1-diphosphate (PR5P) in the nucleotide synthesis pathway. This observation is supported by depletion of sedoheptulose 7-phosphate and glyceraldehyde 3-phosphate that favors ribose 5-phosphate synthesis, and ultimately PR5P formation. NADPH is important for redox balance under stress condition, while PR5P is involved in nucleotide synthesis for DNA repair. These observations coupled with the depleted GSSG and GSH that protect cells against oxidative damage suggest a possible relationship between loss of capsule and oxidative stress in a polyamine-dependent manner. Therefore, DFMO treatment affects polyamine levels, capsule precursor biosynthesis, oxidative stress response, and repair mechanisms in pneumococci (Fig. 5). Overall, the metabolomic signature of DFMO treated Spn provides evidence for the loss of capsule in DFMO-treated cells.

#### D39 metabolites

**Figure 6:**
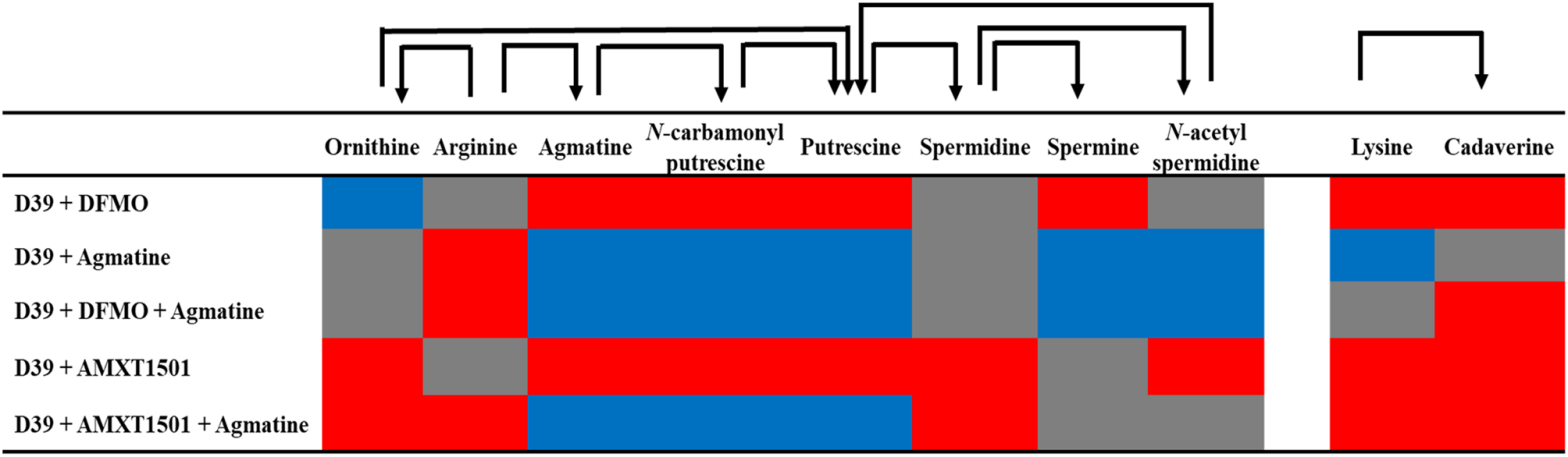
Impact of 1/8 MIC DFMO (34 mM), 1/4 MIC agmatine (20 mM), and 1/2 MIC AMXT 1501 (7.4 µM) on intracellular concentrations of polyamine, precursors, and intermediates in pneumococcal D39. The arrow shows the direction of substrate to intermediate to product. Metabolites in red are depleted, blue are increased, and grey are not significantly impacted.

Following the loss of capsule in DFMO-treated TIGR4 cells and changes in the metabolites that could explain the loss, we carried out DFMO supplementation and measured intracellular polyamines/capsule repeat unit sugars (glucose and glucuronic acid) in D39 serotype, a strain more susceptible to capsule inhibition by DFMO. We further evaluated the impact of treatment with agmatine, a polyamine intermediate known to restore capsule, and polyamine transport inhibitor, AMXT 1501, on intracellular polyamines, their precursors, and intermediates concentrations. Similar to observations made in TIGR4, our results show that DFMO could inhibit the catalysis of arginine to agmatine and further sequential conversion to *N*-carbamoylputrescine, putrescine, and spermine (Fig. 6). Also, there was reduction in the level of lysine and inhibition of its conversion to cadaverine. Accumulation of ornithine suggests that while arginine could be converted to ornithine by arginase, DFMO as an ornithine decarboxylase inhibitor could prevent its further conversion to putrescine. Supplementation of D39 with either only agmatine or in combination with DFMO clearly increases the level of intracellular agmatine, *N*-carbamoylputrescine, putrescine and spermine confirming the essentiality of agmatine in polyamine biosynthesis. As expected, supplementation with agmatine alone did not affect cadaverine production, which is in another pathway. However, presence of DFMO was shown to deplete the level of cadaverine. Similar to the polyamine level pattern with synthesis inhibition by DFMO and restoration by agmatine, AMXT 1501 depleted the levels of polyamines and their intermediates, while the presence of agmatine counteracted AMXT 1501 and replenish these metabolites (Fig. 6).

**Figure 7:**
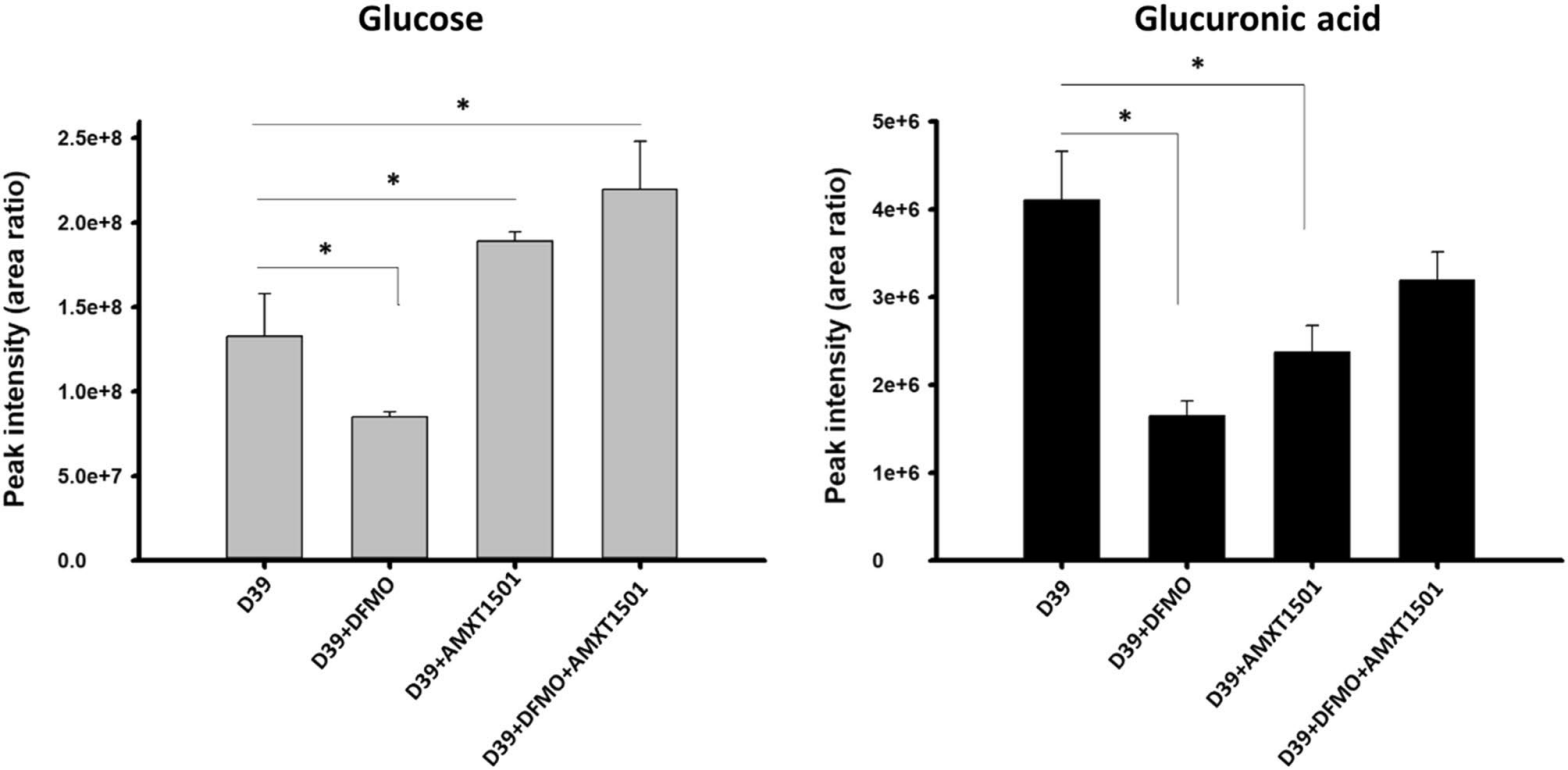
DFMO and AMXT 1501 impact intracellular concentrations of glucose and glucuronic acid in pneumococcal D39. Serotype 2, D39, was treated separately with 1/8 MIC DFMO (34 mM), 1/2 MIC AMXT 1501 (7.4 µM), and in combination, and peak intensities of glucose and glucuronic acid as a measure of their intracellular concentrations were determined. Glucose was significantly reduced in DFMO-treated cells while the presence of AMXT 1501 increases glucose level. Glucuronic acids were significantly depleted in D39 treated separately with DFMO and AMXT 1501 and were moderately reduced when in combination.

Furthermore, specific measurement of intracellular sugars, glucose and glucuronic acid, which are constituents of the capsule repeat unit in D39 shows that DFMO caused depletion of these sugars while AMXT 1501 significantly depletes the glucuronic acid, but increases glucose level (Fig. 7).

### Agmatine reverses the effect of DFMO and AMXT 1501 on CPS 

**Figure 8:**
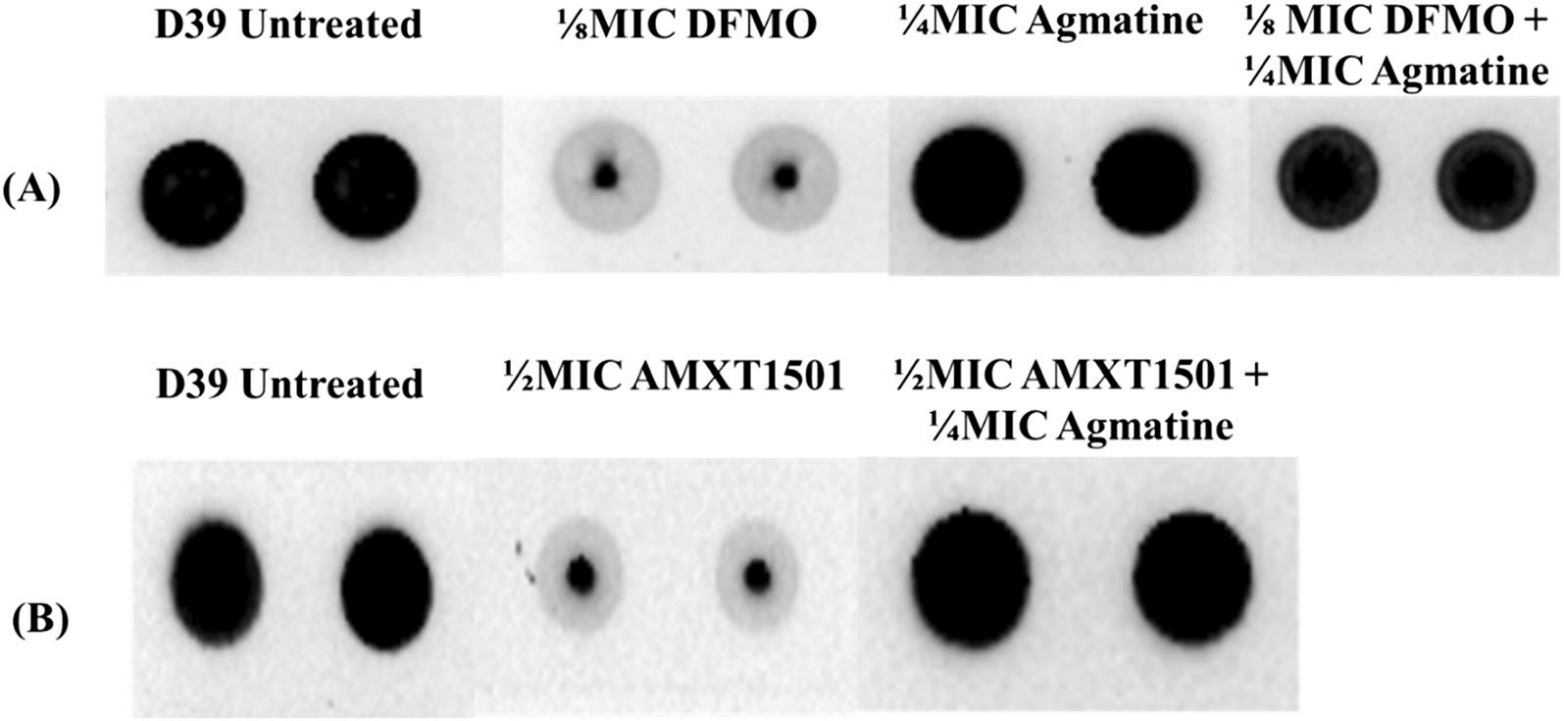
Agmatine reverses the capsule inhibitory effect of (**A**) DFMO, and (**B**) AMXT 1501 in Serotype 2 (D39) pneumococci. Total CPSs isolated from serotype 2 (D39) cultured in THY (untreated), THY supplemented with DFMO (1/8 MIC, 34 mM) alone, agmatine (1/4 MIC, 20 mM) alone, DFMO (1/8 MIC) with agmatine (1/4 MIC), AMXT 1501 (1/2 MIC, 7.4 µM) alone, AMXT 1501 (1/2 MIC, 7.4 µM) and agmatine (1/4 MIC, 20 mM) were spotted on a nitrocellulose membrane. Probing with anti-serotype 2 specific antibody and a horseradish peroxidase (HRP)-conjugated secondary antibody, the membrane was developed with enhanced chemiluminescence (ECL) detection and scanned using a ChemiDoc XRS+ with Image Lab software (Bio-Rad, Hercules, CA, USA). Untreated pneumococcal D39 is shown to be encapsulated. DFMO treatment inhibits the capsule production while agmatine alone does not impact the capsule. Concomitant addition of agmatine in DFMO treated cells restored capsule in D39. Loss of capsule by AMXT 1501 was also fully restored by excess agmatine.

Although DFMO is widely known and reported to target ornithine decarboxylase (ODC), a recent report shows that DFMO could also target arginine decarboxylase that catalyzes the conversion of arginine to agmatine^22^. The metabolic profile of DFMO-treated Spn shows significantly reduced levels of agmatine, the product of ADC activity (Figs. 5 and 6). Furthermore, we recently showed that exogenous supplementation of agmatine (1/4 MIC, 20 mM) restores CPS in the ∆*speA*^18^, a strain that harbors a deletion in the gene encoding ADC. Therefore, agmatine could possibly counteract capsule inhibition by chemicals, which affect the relevant decarboxylases or polyamine pools. Since D39 is more susceptible to DFMO and AMXT 1501 capsule inhibition, we used this strain to determine the impact of exogenous supplementation of agmatine in cells treated with compounds which inhibit polyamine biosynthesis or transport. Addition of agmatine restored capsule in D39 treated with DFMO and AMXT 1501 (Fig. 8).

### DFMO and α-difluoromethylarginine (DFMA) inhibit the decarboxylase activities of arginine decarboxylase

Depletion of intracellular agmatine and cadaverine in DFMO treated pneumococcus suggests that this compound could target the arginine decarboxylase (ADC) that catalyzes the conversion of arginine to agmatine as well as lysine decarboxylase (LDC) that catalyzes conversion of lysine to cadaverine. Although arginine is the preferred substrate, pneumococcal ADC/SP_0916 has ODC and LDC activities^18^. Here, we determine the IC_50_ i.e., the concentration of an inhibitor required to reduce enzymatic activity by 50% for DFMO with recombinant SP_0916 (Table2).

**Table 2:** DFMO and DFMA IC_50_ with the Michaelis–Menten constant (*K*_*m*_) of the enzyme for different decarboxylase substrates.

Substrate	DFMO IC_50_ (mM)	DFMA IC_50_ (µM)	*K*_*m*_ (mM)*
Agmatine	75.1 ± 5.5	1.1 ± 0.1	0.11 ± 0.02
Putrescine	26.6 ± 4.1	40.5 ± 11.6	3.55 ± 0.28
Cadaverine	8.2 ± 0.2	56.6 ± 11.9	1.61 ± 0.28

*Previously reported^18^.

**Figure 9:**
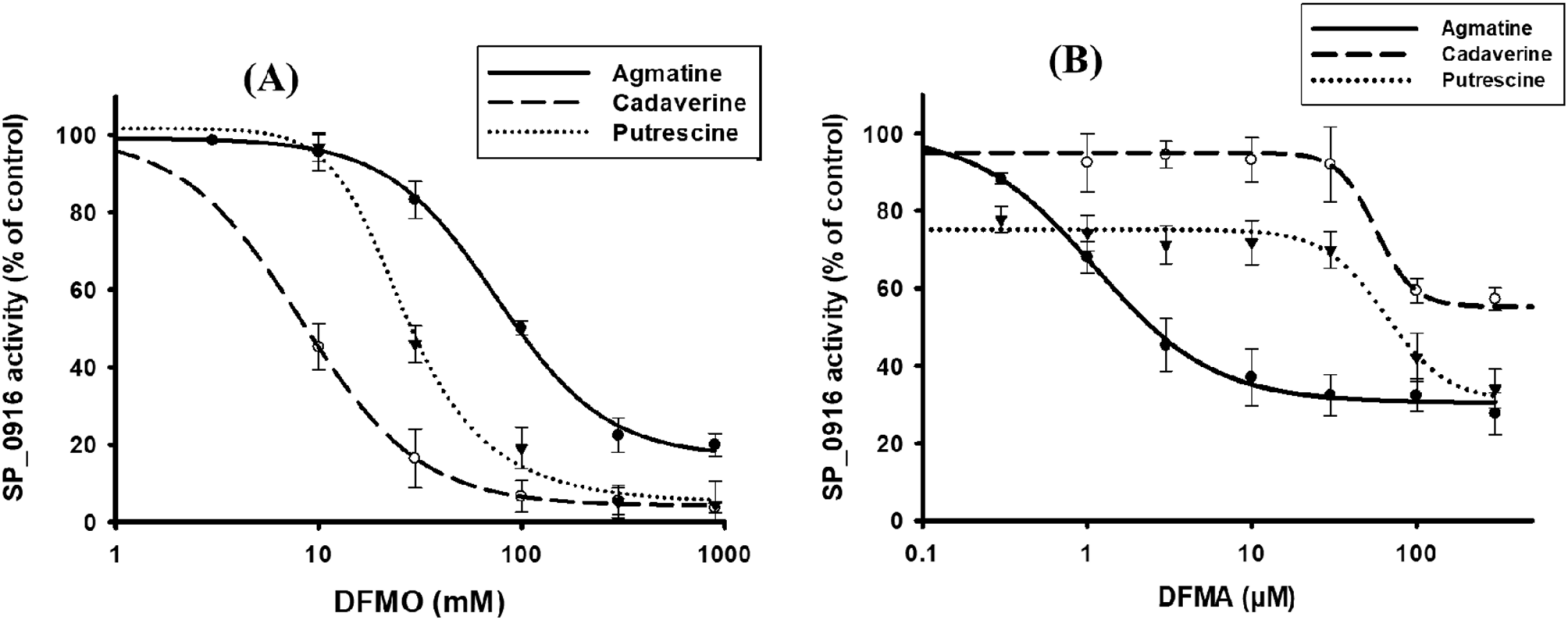
DFMO (**A**) and DFMA (**B**) inhibit decarboxylase activities of ADC. We determined DFMO/DFMA IC_50_ values for the following reactions catalyzed by ADC: arginine to agmatine, lysine to cadaverine, and ornithine to putrescine. Enzyme activity without inhibitor is referred to as a control activity. LCMS based estimation of IC_50_ was determined by varying the concentration of the indicated inhibitor (a) DFMO or (b) DFMA and measuring the decarboxylase activity. Sigma Plot v. 12 was used to fit the curve through the points and IC_50_ values were interpolated from the fitted curve. Our data shows that DFMO IC_50_ for agmatine (75.1 ± 5.5 mM) > putrescine (26.6 ± 4.1 mM) > cadaverine (8.2 ± 0.2 mM) while IC_50_ of α-difluoromethylarginine (DFMA) that is specific for arginine decarboxylase show that cadaverine (56.6 ± 11.9 µM) > putrescine (40.5 ± 11.6 µM) > agmatine (1.1 ± 0.1 µM).

Our data shows that DFMO IC_50_ for agmatine (75.1 ± 5.5 mM) > putrescine (26.6 ± 4.1 mM) > cadaverine (8.2 ± 0.2 mM) (Table 2 and Fig. 9A). This result indicates that DFMO inhibits all the decarboxylase activities of ADC, albeit, at high concentration. Furthermore, we evaluated the IC_50_ of α-difluoromethylarginine (DFMA) that is a specific inhibitor of ADC. IC_50_ of DFMA with cadaverine (56.6 ± 11.9 µM) > putrescine (40.5 ± 11.6 µM) > agmatine (1.1 ± 0.1 µM) (Table 2 and Fig. 9B). *Km* of ADC with different substrates^18^ are also shown.

## Discussion

DFMO is a suicide inhibitor of eukaryotic ornithine decarboxylase (ODC) and has been studied extensively for its antiproliferative effects in different diseases involving uncontrolled cell growth. Polyamine synthesis in pneumococci has been shown to have a significant effect on capsule expression in pneumococci^17,18,24^. In this study, we evaluated the effect of DFMO as a polyamine synthesis inhibitor in pneumococci, as well as the impact and synergistic effect of AMXT 1501 as polyamine transport inhibitor. Our findings indicate that DFMO inhibits CPS in multiple pneumococcal serotypes (Fig. 3A) while AMXT 1501 appears to inhibit in only serotype 2, (Fig. 3B). Capsule biosynthesis generally involves polymerization of approximately 2–8 simple and/or nucleotide sugars depending on the strain^31^. Metabolomic analysis of DFMO-treated serotypes showed that DFMO resulted in depletion of 3 out of the 4 sugars in the repeat unit of serotype 4 capsule examined in this study, and the 2 sugars in the repeat units of serotype 2. Metabolomics analysis also identified pathways such as Leloir, glycolysis, and nucleotide sugar biosynthesis that produce these repeat unit sugars being negatively impacted by DFMO (Fig. 5). These results are in agreement with our previous report on the impact of inhibition of polyamine synthesis by deletion of ADC in serotype 4^24^. The net effect of the identified changes in the metabolome upon exposure to DFMO ultimately translate to reduced availability of precursors for CPS. Metabolic changes such as depleted levels of uracil^32^ or acetyl-CoA^33^ that resulted in loss of capsule have been reported. However, these changes are not serotype independent. Indeed, this report suggests that polyamine synthesis modulation by DFMO targets serotypes 2 and 4.

The extent of capsule inhibition by DFMO appears to depend on the type of CPS synthesis pathway. DFMO is more effective against the serotypes that utilize Wzy-dependent pathway, which is responsible for the CPS synthesis in all pneumococci with the exception of only two strains^23^. WU2, one of the two strains that follow unconventional synthase pathway for CPS synthesis has the least amount of capsule loss observed in this study (Fig. 3A). Our data also indicates subtle differences in serotype susceptibility of CPS inhibition by DFMO, as serotype 2 CPS was inhibited with relatively lower concentrations of DFMO compared to other strains. In addition, we examined possible synergism between DFMO and the polyamine transport inhibitor, AMXT 1501. Contrary to what was reported in the eukaryotic system^9,10,34^, no synergism was observed between the two compounds in pneumococci with respect to capsule inhibition. AMXT 1501 at 1/2 MIC had no impact on CPS in serotypes 4, 3 and 19F, although, serotype 2 CPS and polyamine synthesis were inhibited by this eukaryotic polyamine transport inhibitor. The mechanisms that render serotype 2 CPS synthesis highly sensitive to modulation of polyamine homoeostasis by chemical manipulation are yet to be established. DFMO inhibits capsule biosynthesis in all the tested pneumococcal serotypes at a sub-lethal dose (high mM concentration). The safe dosage of DFMO for use in human is around 1.0 g/m^2^/day (~ 1 to 2 mM)^35^. Even in D39 that is more susceptible to DFMO inhibition, the concentration that inhibits CPS is 34 mM. Although the DFMO concentration was high, it allowed us to perform exogenous supplementation assays with polyamine intermediates that can compete with this competitive inhibitor of ODC. In this study DFMO was a useful tool to dissect the mechanisms at the intersection of altered polyamine synthesis and inhibition of CPS. Initial assessment of additional polyamine inhibitor, DFMA, shows that it could inhibit at a micromolar range (Fig. 9), however, its full characterization is beyond the scope of this work. Furthermore, we previously reported that agmatine, the precursor for putrescine is critical for CPS synthesis^18^. Here, we have shown that agmatine can reverse DFMO and AMXT 1501 inhibition of CPS in serotype 2. Taken together, DFMO/Agmatine represent a CPS OFF/ON switch that can help identify novel CPS regulatory mechanisms governed by intracellular polyamine levels. Thus, DFMO and AMXT 1501 serve as important tools to dissect the mechanisms at the intersection of altered polyamine synthesis/transport and inhibition of CPS.

Apart from reduced agmatine level, additional signatures of DFMO inhibition identified in this study include the depletion of THF and methionine which is consistent with report on the effect of DFMO in reducing thymine pool and methionine in cancer cell^36^. Reduced putrescine and spermidine are in accordance with the functional role of DFMO in blocking the catalysis of ornithine to putrescine, and subsequently to spermidine. The *N*-acetylspermidine level is also low in DFMO-treated TIGR4 but there was no change in the level of *N*-acetylspermine indicating that the synthesis and accumulation of spermine is likely being favored over its catabolic degradation. However, spermine was depleted in D39 treated DFMO. Studies have shown that DFMO does not impact spermine concentration in eukaryotic cells^6,37^. The reasons for accumulation/depletion of spermine in the two strains are not known at present. Concurrent acetylation of spermidine and spermine could account for the depletion of spermine in D39 similar to *Bacillus subtilis* where the *pai* operon possess *N*-acetyltransferase activity towards both spermidine and spermine^38^. Accumulation of spermine in TIGR4 could be part of pneumococcal defense against oxidative stress as spermine is a known antioxidant^39,40^. DFMO is capable of inducing stress and the catabolism of spermine to spermidine also contributes to reactive oxygen species in the form of hydrogen peroxide generation which may cause additional stress to the system^4,15^.

Since polyamines are known antioxidants^41,42^, depletion of spermidine, putrescine, and cadaverine will most likely cause elevated levels of oxidants. We hypothesized that reduced levels of polyamines in response to DFMO treatment shuts down energy consuming capsule production and redirects carbon flux through PPP to generate NADPH to combat oxidative stress. An elevated level of reduced glutathione (GSH) and depleted level of oxidized glutathione (GSSG) in DFMO treated cells have been reported earlier^43,44^. Here, we observed depleted levels of GSH and GSSG in DFMO-treated cells. The glutathione system is important for redox homeostasis. Therefore, these results indicate high levels of reactive oxygen species and stress due to loss of intracellular polyamines in DFMO-treated pneumococci. The significantly higher levels of 5-phospho-α-d-ribose 1-diphosphate (PRPP), the key molecule in nucleotide biosynthesis, suggests that an activated repair mechanism may be triggered in response to DFMO to maintain the physiology of the pneumococcal cells.

In recent years, evidence shows that the DFMO inhibitory effect extends beyond impeding ODC activity and includes arginine decarboxylase (ADC) in the alternative pathway for polyamine biosynthesis^22^. In addition, DFMO was shown to inhibit the activity of arginase in a colon cancer study and a recent clinical trial for the early treatment of Alzheimer’s disease^45,46^. The significant reduced level of agmatine in the DFMO treated cells in this study confirmed that DFMO, indeed, inhibits arginine decarboxylase activity in bacteria. Initial characterization of the impact of DFMO on amino acid decarboxylase activities shows that it could inhibit ornithine, arginine, and lysine decarboxylation reactions (Fig. 9). However, current annotations of pneumococcal genomes are still missing specific genes that encode ODC and LDC enzymes. *S. pneumoniae* TIGR4 genome has two additional genes, SP_0166 and SP_0920 that are annotated with PLP dependent decarboxylase activities. Biochemical characterization of enzymes encoded by these two genes is warranted for the annotation of polyamine biosynthesis pathway in Spn.

In conclusion, we show that chemical inhibition of polyamine synthesis and transport with DFMO and AMXT 1501 results in reduced CPS which was restored by agmatine, similar to earlier report using a gene deletion approach. These results demonstrate that polyamine mediated regulation of capsule is conserved between genetic and chemical modulation of polyamine pathways. Furthermore, mechanisms of CPS regulation are shared between altered polyamine synthesis and transport presumably because both affect intracellular polyamine pools. Since polyamine biosynthesis and transport genes are well conserved across multiple pneumococcal serotypes, understanding polyamine-CPS dynamics could help identify novel-serotype independent antimicrobial targets for pneumococci eradication. Polyamine synthesis is found in both pneumococci and their human host, but CPS is specific to the pathogens. Deconvoluting the DFMO-AMXT 1501/agmatine regulation of CPS can help identify specific targets in Spn for therapeutic intervention to limit the spread of this pathogen. This work will serve as a pivot for establishing polyamines as global regulators of pneumococcal virulence, and validates targeting of polyamine metabolic pathways as novel therapies for pneumococcal infections.

## Supplementary Information

Supplementary Information.

## Data Availability

The data that support the findings of this study are available in the manuscript and supplementary information.
