# Supplementary material for: Difluoromethylornithine (DFMO) and AMXT 1501 inhibit capsule biosynthesis in pneumococci
Source: Sci Rep. 2022 Jul 12;12:11804. doi: 10.1038/s41598-022-16007-7 (PMC9276676; doi:10.1038/s41598-022-16007-7)
Supplement: Supplementary file 1 — Supplementary Information. [file 41598_2022_16007_MOESM1_ESM.docx]

**Supplementary Table 1**: Significant fold changes in the metabolites of *S. pneumoniae* TIGR4 in response to DFMO.

|  | **Metabolites** | **TIGR4+DFMO /TIGR4** | **FDR** | **Functions** |
| --- | --- | --- | --- | --- |
| 1 | Spermidine | -3.1 | 0.00028 | Polyamine |
| 2 | Putrescine | -1.9 | 0.05200 | Polyamine |
| 3 | Spermine | 26.6 | 0.00035 | Polyamine |
| 4 | Cadaverine | -1.5 | 0.01000 | Polyamine |
| 5 | Arginine | 1.1 | 0.04623 | Polyamine biosynthesis precursor |
| 6 | Ornithine | -1.3 | 0.05700 | Polyamine biosynthesis precursor |
| 7 | Lysine | -1.7 | 0.00070 | Polyamine biosynthesis precursor |
| 8 | Methionine | -1.8 | 0.00617 | Polyamine biosynthesis intermediate |
| 9 | Agmatine | -2.0 | 0.00012 | Polyamine biosynthesis intermediate |
| 10 | 5,10-methylenetetrahydrofolate | 4.4 | 0.00237 | Polyamine biosynthesis intermediate |
| 11 | Tetrahydrofolate | -1.4 | 0.01182 | Polyamine biosynthesis intermediate |
| 12 | *S*-adenosylmethionine | 64.2 | 0.00151 | Polyamine biosynthesis intermediate |
| 13 | *N*-Acetylspermidine | -1.6 | 0.00004 | Polyamine catabolism intermediate |
|  |  |  |  |  |
| 14 | Galactose 1-phosphate | -16.8 | 0.03440 | Leloir Pathway |
| 15 | UDP-Glucose | -66.5 | 0.00004 | Leloir Pathway |
| 16 | UDP-Galactose | -66.5 | 0.00004 | Leloir Pathway/Capsule precursor |
|  |  |  |  |  |
| 17 | Glucose 6-phosphate | 10.6 | 0.00017 | Glycolysis/PPP |
| 18 | Fructose 6-phosphate | -16.8 | 0.03440 | Glycolysis |
| 19 | Glyceraldehyde 3-phosphate | -308.6 | 0.00001 | Glycolysis |
| 20 | Pyruvate | -1.74 | 0.00028 | Glycolysis |
| 21 | NADH | -358.8 | 0.00015 | Glycolysis |
|  |  |  |  |  |
| 22 | Glucosamine 6-phosphate | -21.0 | 0.00031 | Nucleotide sugar biosynthesis |
| 23 | UDP-*N*-acetylglucosamine | -18.9 | 0.00107 | Nucleotide sugar biosynthesis |
|  |  |  |  |  |
| 24 | UDP-*N*-acetylgalactosamine | -18.9 | 0.00107 | Capsule precursor |
| 25 | UDP-*N*-acetylmannosamine | -18.9 | 0.00107 | Capsule precursor |
| 26 | UDP-*N*-acetylfucosamine | 2.3 | 0.01846 | Capsule precursor |
| 27 | Fucose | -1.3 | 0.00809 | Capsule precursor |
| 28 | UDP-*N*-acetylmuramic acid | -259.2 | 0.00007 | Peptidoglycan biosynthesis |
|  |  |  |  |  |
| 29 | UMP | -2.1 | 0.00277 | Pyrimidine synthesis |
| 30 | UDP | -53.1 | 0.00031 | Pyrimidine synthesis |
|  |  |  |  |  |
| 31 | 6-Phosphogluconate | 7.8 | 0.00062 | Pentose phosphate pathway |
| 32 | Sedoheptulose 7-phosphate | -13.5 | 0.00126 | Pentose phosphate pathway |
| 33 | NADPH | 25.1 | 0.00191 | Pentose phosphate pathway |
|  |  |  |  |  |
| 34 | 5-phospho-α-D-ribose 1-diphosphate (PRPP) | 3.8 | 0.04358 | Nucleotide synthesis |
| 35 | GSH | -23.2 | 0.00578 | Stress Response |
| 36 | GSSG | -41.3 | 0.00504 | Stress Response |

**Supplementary Table 2**: List of non-significant metabolites identified during DFMO treatment of *S. pneumoniae* TIGR4

| 1 | 6-phosphogluconolactone |
| --- | --- |
| 2 | Acetate |
| 3 | Acetyl-CoA |
| 4 | Erythrose 4-phosphate |
| 5 | Fructose |
| 6 | Galactose |
| 7 | Glucose |
| 8 | Glycine |
| 9 | *L*-aspartate |
| 10 | *L*-glutamine |
| 11 | *N*-AcetylSpermine |
| 12 | Phosphoenol pyruvate |
| 13 | Ribose 5-phosphate |
| 14 | Serine |
| 15 | UDP-GlcNAc |
| 16 | UTP |
| 17 | Xylulose 5-phosphate |

**Supplementary Table 3**: Significant fold changes in the metabolites of *S. pneumoniae* D39 in response to DFMO, Agmatine, AMXT 1501 treatments alone and in combination.

| Metabolites | D39+DFMO  /D39 | D39+Agmatine  /D39 | D39+DFMO+Agmatine  /D39 | D39+AMXT  /D39 | D39+Agmatine+AMXT  /D39 |
| --- | --- | --- | --- | --- | --- |
| Arginine | 0.1 (0.158) | -0.4 (0.049) | -0.6 (0.006) | -0.1 (0.546) | -0.5 (0.008) |
| Lysine | -0.2 (0.018) | 0.1 (0.048) | -0.1 (0.377) | -0.5 (3.48E-04) | -0.5 (0.001) |
| Ornithine | 0.4 (0.004) | 0.1 (0.149) | -0.1 (0.637) | -0.5 (0.004) | -0.7 (0.002) |
| Agmatine | -1.3 (2.13E05) | 10.2 (1.61E-06) | 9.8 (2.11E-09) | -1.5 (0.006) | 9.4 (3.54E-06) |
| Cadaverine | -0.8 (0.004) | -0.3 (0.204) | -0.5 (0.041) | -1.2 (4.61E-04) | -1.8 (3.63E-04) |
| Putrescine | -0.4 (0.015) | 3.3 (0.004) | 3.2 (0.001) | -1.4 (0.001) | 2.9 (3.58E-04) |
| Spermidine | 0.3 (0.309) | 0.6 (0.283) | 0.2 (0.552) | -5.5 (0.006) | -4.5 (0.007) |
| Spermine | -0.7 (0.002) | 1.4 (3.30E-04) | 0.5 (2.86E-04) | -0.3 (0.455) | 0.8 (0.067) |
| *N*-carbamoyl putrescine | -1.6 (0.026) | 9.6 (1.58E-05) | 9.3 (1.02E-04) | -1.8 (0.016) | 8.6 (1.80E-04) |
| *N*-acetyl spermidine | -0.6 (0.094) | 0.8 (0.009) | 0.7 (0.094) | -1.6 (0.022) | -0.6 (0.102) |

***** Individual treatment’s *p*-value is enclosed in the bracket. Significant metabolite changes between untreated and treated wild type pneumococci D39 was identified using student’s *t*-test at a *p*-value of < 0.05.

**Supplementary Table 4:** Growth rates and maximal O.D of pneumococcal serotype TIGR4, WU2, D39 and EF3030.

|  | **DFMO (µg/µL)** | **Growthrate** | **Max OD** |
| --- | --- | --- | --- |
| VC TIGR4 | 0.0 | 0.049 | 1.590 |
| 1 | 100.0 | 0.000 | 0.000 |
| 2 | 50.0 | 0.000 | 0.000 |
| 3 | 25.0 | 0.052 | 1.280 |
| 4 | 12.5 | 0.056 | 1.560 |
| 5 | 6.3 | 0.055 | 1.635 |
| 6 | 3.1 | 0.056 | 1.654 |
| 7 | 1.6 | 0.057 | 1.695 |
| 8 | 0.8 | 0.056 | 1.657 |
| 9 | 0.4 | 0.056 | 1.619 |
| 10 | 0.2 | 0.055 | 1.583 |
|  |  |  |  |
| VC WU2 | 0.0 | 0.047 | 0.459 |
| 1 | 100.0 | 0.000 | ---- |
| 2 | 50.0 | 0.000 | ---- |
| 3 | 25.0 | 0.046 | 0.303 |
| 4 | 12.5 | 0.048 | 0.696 |
| 5 | 6.3 | 0.048 | 0.652 |
| 6 | 3.1 | 0.050 | 0.650 |
| 7 | 1.6 | 0.046 | 0.575 |
| 8 | 0.8 | 0.047 | 0.548 |
| 9 | 0.4 | 0.047 | 0.509 |
| 10 | 0.2 | 0.046 | 0.497 |
|  |  |  |  |
| VC D39 | 0.0 | 0.037 | 1.593 |
| 1 | 100.0 | 0.000 | 0.000 |
| 2 | 50.0 | 0.000 | 0.000 |
| 3 | 25.0 | 0.029 | 0.985 |
| 4 | 12.5 | 0.036 | 1.613 |
| 5 | 6.3 | 0.037 | 1.647 |
| 6 | 3.1 | 0.039 | 1.642 |
| 7 | 1.6 | 0.040 | 1.648 |
| 8 | 0.8 | 0.018 | 1.615 |
| 9 | 0.4 | 0.019 | 1.573 |
| 10 | 0.2 | 0.025 | 1.555 |
|  |  |  |  |
| VC EF3030 | 0.0 | 0.037 | 1.426 |
| 1 | 100.0 | 0.000 | 0.000 |
| 2 | 50.0 | 0.000 | 0.000 |
| 3 | 25.0 | 0.040 | 1.319 |
| 4 | 12.5 | 0.030 | 1.513 |
| 5 | 6.3 | 0.035 | 1.538 |
| 6 | 3.1 | 0.036 | 1.517 |
| 7 | 1.6 | 0.037 | 1.495 |
| 8 | 0.8 | 0.037 | 1.485 |
| 9 | 0.4 | 0.021 | 1.458 |
| 10 | 0.2 | 0.035 | 1.413 |


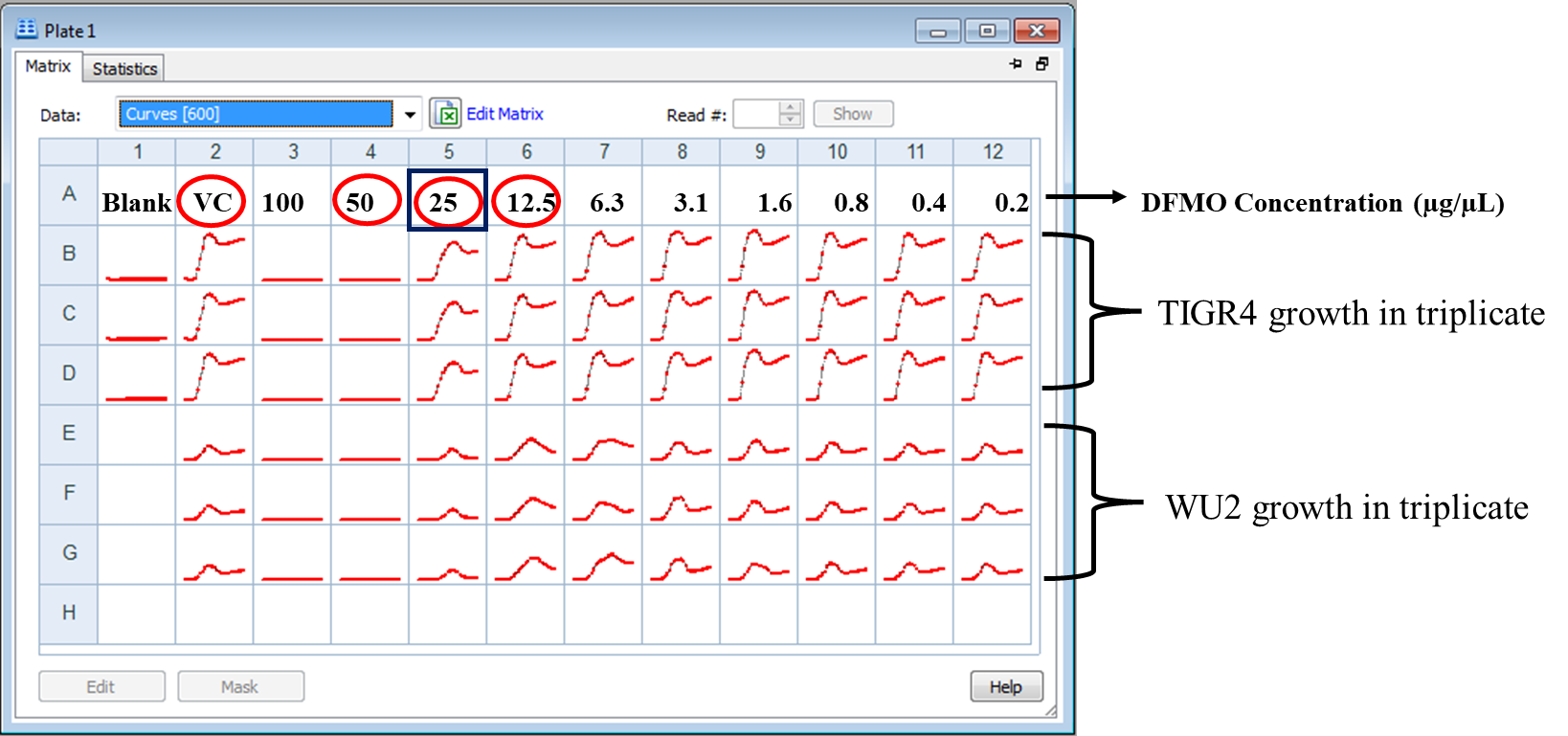


**Supplementary Figure 1.** Determination of minimum inhibition concentration of DFMO in pneumococcal serotypes TIGR4 and WU2. The starting inoculum was ~10^5^ CFU/mL. While blank was bacteria culture media without pneumococcal inoculum, the viability control (VC) contained individual serotypes without DFMO. Our data show that the minimum inhibitory concentration for both serotypes is 25 µg/µL (137 mM).


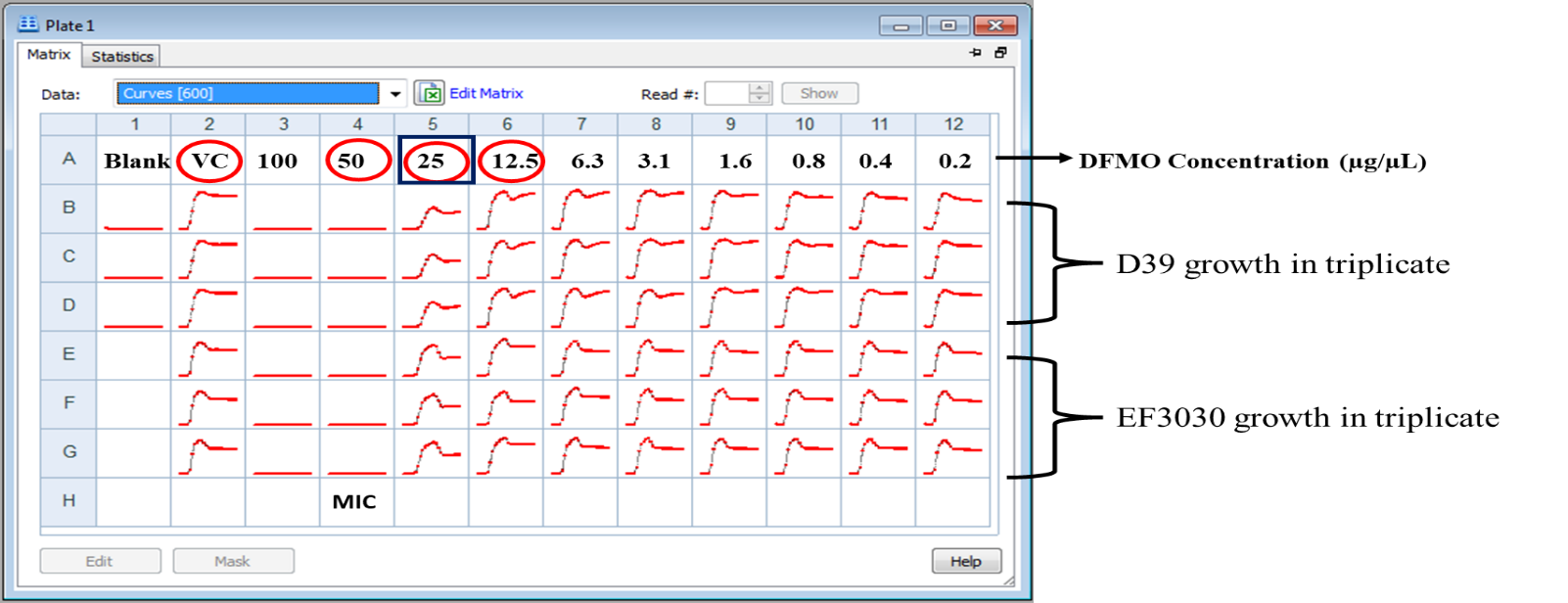


**Supplementary Figure 2.** Determination of minimum inhibition concentration of DFMO in pneumococcal serotypes D39 and EF3030. The starting inoculum was ~10^5^ CFU/mL. While blank was bacteria culture media without pneumococcal inoculum, the viability control (VC) contained individual serotypes without DFMO. Our data show that the minimum inhibitory concentration for both serotypes is 25 µg/µL (137 mM).
